# Corneal optical densitometry in transparent corneas and its correlations with corneal higher-order aberrations

**DOI:** 10.1186/s12886-025-04583-x

**Published:** 2026-01-19

**Authors:** Ji Sun, Bin Xu, Ling Zhou, Zhangyi Li, Jiayu Zhang, Wenjuan Wan, Can Li

**Affiliations:** 1 Chongqing Key Laboratory of Ophthalmology and Chongqing Eye Institute, The First Affiliated Hospital of Chongqing Medical University, Chongqing, 400016 China; 2 Chongqing Eye and Vision Care Hospital, Chongqing, 400000 China

**Keywords:** Corneal optical densitometry, Corneal higher-order aberrations, Spherical aberration, Visual quality, Corneal transparency

## Abstract

**Purpose:**

This study aimed to evaluate correlations between corneal optical densitometry (COD) and corneal higher-order aberrations (HOAs).

**Design:**

A prospective cross-sectional study.

**Methods:**

A total of 67 participants were enrolled. The Pentacam quantified total COD and corneal HOAs. Coma and trefoil were described using the root mean square (RMS). HOAs measurements, including total HOA, spherical aberration (Z40), vertical coma (Z3-1), horizontal coma (Z31), oblique trefoil (Z3-3), horizontal trefoil (Z33), coma RMS (Z3 ± 1 RMS), and trefoil RMS (Z3 ± 3 RMS) of the total, anterior, and posterior corneas, were calculated for both the central 4.0 mm diameter and the central 6.0 mm diameter zones.

**Results:**

In the central 4.0 mm diameter zone, total COD exhibited significant positive correlations with HOA, Z40, and Z3 ± 1 RMS of the total cornea, HOA and Z40 of the anterior cornea, as well as Z40 and Z31 of the posterior cornea; in the 6.0 mm zone, total COD was positively correlated with HOA, Z40, and Z3 ± 1 RMS of the total cornea, HOA of the anterior cornea, as well as Z40, Z3-1, Z31, and Z3 ± 1 RMS of the posterior cornea (*P* < 0.05 for each one). Age and COD showed a statistically positive correlation (r_s_ = 0.350, *P* = 0.004).

**Conclusions:**

An increase in COD may result in elevated levels of corneal HOAs, particularly including total HOA, spherical aberration, and coma. This increase could potentially compromise the sphericity, symmetry, and regularity of the cornea due to age-related changes in corneal microstructure and composition, even in clinically transparent corneas.

**Supplementary Information:**

The online version contains supplementary material available at 10.1186/s12886-025-04583-x.

## Introduction

The demand for high-quality visual images has increased due to the improvement in living standards. Nevertheless, blurry retinal images and decreased visual performance can be caused by optical aberrations, which can be mathematically expressed and classified by a series of Zernike polynomials [1]. Approximately 10% of the total wavefront aberrations are attributed to HOAs, which are dominated by coma-like aberrations (vertical/horizontal coma, oblique/horizontal trefoil) and spherical aberration [2, 3]. As the primary optical component of the human eye, the cornea accounts for approximately 70% of the total optical power and contributes about 90% to ocular aberrations [4, 5]. Previous studies have shown that corneal HOAs can explain decreases in contrast sensitivity and visual acuity in both normal eyes and those undergoing ocular surgery [6], thus allowing clinicians to gain a better understanding of patients’ visual performance.

Due to its unique structure, the healthy human cornea transmits a significant proportion of visible light and is optically transparent [7]. The subjective assessment of corneal transparency using biomicroscopes such as slit lamp microscopy has been employed for decades [8]. However, corneal optical densitometry or corneal optical density (COD), a novel concept introduced in recent years, can objectively assess the transparency and optical density of the cornea [9–11]. This measurement can be conducted through a Pentacam Scheimpflug analysis system (Oculus Optikgeräte GmbH, Wetzlar, Germany). In clinical practice, the Pentacam system utilizes a rotating lens to acquire high-resolution images noninvasively; it provides precise COD values and measurements of corneal HOAs in different corneal regions [12–14]. 

This study aimed to investigate the potential influence of COD on corneal HOAs and visual quality by analyzing correlations between total COD values and corneal HOAs.

## Materials and methods

This is a prospective cross-sectional study. In the preliminary experiment, Pearson’s correlation tests were conducted to quantify the exact correlations between total COD and corneal HOAs, with the correlation coefficient r serving as the primary evaluation metric. The results indicated that r was 0.115. A two-sided test was performed with α set at 0.05 and Power (test efficiency) set at 90%. The sample size calculated using PASS 15 software was *N* = 58. Considering a 10% dropout and refusal rate, at least 65 subjects were required. Ultimately, a total of 67 participants were recruited at the First Affiliated Hospital of Chongqing Medical University, China, between 2021 and 2024. This study excluded participants who had experienced keratopathy, glaucoma, ocular surgery, or wearing contact lenses in the previous month and those who did not adequately cooperate with the Pentacam system. For each eye, an experienced examiner measured corneal HOAs and COD using the same Pentacam. COD and HOAs analysis is provided as part of the standard software of the Pentacam Scheimpflug device. The measurements were conducted following standardized procedures and under controlled dim-light conditions. Coma and trefoil aberrations were described using the root mean square (RMS). Since there was a strong correlation between the subject’s right eye and left eye optical densitometry results [15], only the right eye was evaluated. All images enrolled in this study were confirmed to be of acceptable quality.

Simultaneously, COD was measured using the same Pentacam. COD measurements with the Pentacam are divided into four concentric annular zones at the apex of the cornea: 0–2 mm, 2–6 mm, 6–10 mm, and 10–12 mm, as well as three layers in depth: the anterior layer (superficial 120 μm), the posterior layer (posterior 60 μm), and the central layer (located between the anterior and the posterior layers) [16, 17]. Moreover, Pentacam provides a comprehensive measurement of optical densitometry across the entire cornea. In this study, the total COD value was selected as the primary parameter for analysis. The Pentacam can quantify backscattered light and COD, which assigns backscatter a score from 0 to 100 representing grayscale units (GSU). A higher score suggests higher backscatter and decreased transparency of the cornea [18]. In the meantime, the corneal HOAs measurements with the Pentacam included the following parameters: HOA (total), spherical aberration (Z40 (total)), vertical coma (Z3-1 (total)), horizontal coma (Z31 (total)), oblique trefoil (Z3-3 (total)), horizontal trefoil (Z33 (total)), coma aberration RMS (Z3 ± 1 RMS (total)), and trefoil aberration RMS (Z3 ± 3 RMS (total)) of the total corneas; HOA (anterior), spherical aberration (Z40 (anterior)), vertical coma (Z3-1 (anterior)), horizontal coma (Z31 (anterior)), oblique trefoil (Z3-3 (anterior)), horizontal trefoil (Z33 (anterior)), coma aberration RMS (Z3 ± 1 RMS (anterior)), and trefoil aberration RMS (Z3 ± 3 RMS (anterior)) of the anterior corneas; HOA (posterior), spherical aberration (Z40 (posterior)), vertical coma (Z3-1 (posterior)), horizontal coma (Z31 (posterior)), oblique trefoil (Z3-3 (posterior)), horizontal trefoil (Z33 (posterior)), coma aberration RMS (Z3 ± 1 RMS (posterior)), and trefoil aberration RMS (Z3 ± 3 RMS (posterior)) of the posterior corneas. HOAs were calculated for both the central 4.0 mm diameter and the central 6.0 mm diameter zones, which represent the main central optical region and are widely used to assess wavefront aberrations.

### Statistical analysis

We used the SPSS software (version 25.0, IBM Corp.) to analyze the measurement data. For the continuous data, the Shapiro-Wilk test was used to evaluate the normal distribution. Descriptive analysis was employed to assess the acquired data, including the mean ± standard deviation (SD) for results with a normal distribution and the median (quartile range) for those not having a normal distribution. Besides, we used the Spearman rank correlation to determine the exact relationship between total COD and age and the correlations between total COD and corneal HOAs when the data followed a non-normal distribution. A *P* < 0.05 was considered statistically significant.

## Results

The study included 67 participants with 67 eyes, with a mean age of 64.1 ± 7.4 years. The total COD values exhibited a non-normal distribution, with a median (quartile range) value of 15.3 (14.1 to 16.9) GSU. Furthermore, corneal HOAs values that followed a normal distribution were expressed as mean ± SD and were shown in Table 1, while those with a non-normal distribution were described using the median and quartile range, as presented in Table 2.

**Table 1:** The distribution of corneal higher-order aberrations with a normal distribution

Aberrations (µm)	Mean ± SD	
	The 4.0 mm zone	The 6.0 mm zone
Z40 (total)	0.071 ± 0.041	0.318 ± 0.112
Z3-1 (total)	0.041 ± 0.094	0.098 ± 0.256
Z31 (total)	-0.008 ± 0.061	0.024 ± 0.139
Z3-3 (total)	-0.010 ± 0.052	-
Z33 (total)	-	0.005 ± 0.126
HOA (anterior)	0.185 ± 0.065	-
Z40 (anterior)	0.087 ± 0.039	0.329 ± 0.096
Z3-1 (anterior)	0.053 ± 0.095	0.061 ± 0.234
Z31 (anterior)	-0.018 ± 0.057	-0.003 ± 0.125
Z3-3 (anterior)	-0.015 ± 0.048	-
Z33 (anterior)	-	0.015 ± 0.116
HOA (posterior)	0.060 ± 0.011	0.183 ± 0.031
Z40 (posterior)	-0.032 ± 0.010	-0.123 ± 0.038
Z3-1 (posterior)	-0.011 ± 0.021	0.021 ± 0.062
Z31 (posterior)	0.009 ± 0.017	0.026 ± 0.027
Z3 ± 1 RMS (posterior)	0.027 ± 0.014	-
Z3-3 (posterior)	-	-0.012 ± 0.044
Z33 (posterior)	0.007 ± 0.021	-0.013 ± 0.040
Z3 ± 3 RMS (posterior)	-	0.056 ± 0.026

*Significant

**Table 2:** The distribution of corneal higher-order aberrations with a non-normal distribution

Aberrations (µm)	Median (quartile range)	
	The 4.0 mm zone	The 6.0 mm zone
HOA (total)	0.179 (0.134 to 0.217)	0.514 (0.414 to 0.620)
Z3 ± 1 RMS (total)	0.091 (0.049 to 0.140)	0.247 (0.143 to 0.320)
Z3-3 (total)	-	-0.119 (-0.208 to 0.031)
Z33 (total)	0.009 (-0.013 to 0.051)	-
Z3 ± 3 RMS (total)	0.057 (0.041 to 0.081)	0.168 (0.117 to 0.268)
HOA (anterior)	-	0.503 (0.407 to 0.583)
Z3 ± 1 RMS (anterior)	0.097 (0.052 to 0.148)	0.214 (0.136 to 0.292)
Z3-3 (anterior)	-	-0.075 (-0.170 to 0.021)
Z33 (anterior)	-0.006 (-0.030 to 0.032)	-
Z3 ± 3 RMS (anterior)	0.051 (0.037 to 0.081)	0.161 (0.096 to 0.224)
Z3 ± 1 RMS (posterior)	-	0.055 (0.037 to 0.081)
Z3-3 (posterior)	0.006 (-0.014 to 0.021)	-
Z3 ± 3 RMS (posterior)	0.025 (0.019 to 0.035)	-

*Significant

**Fig. 1:**
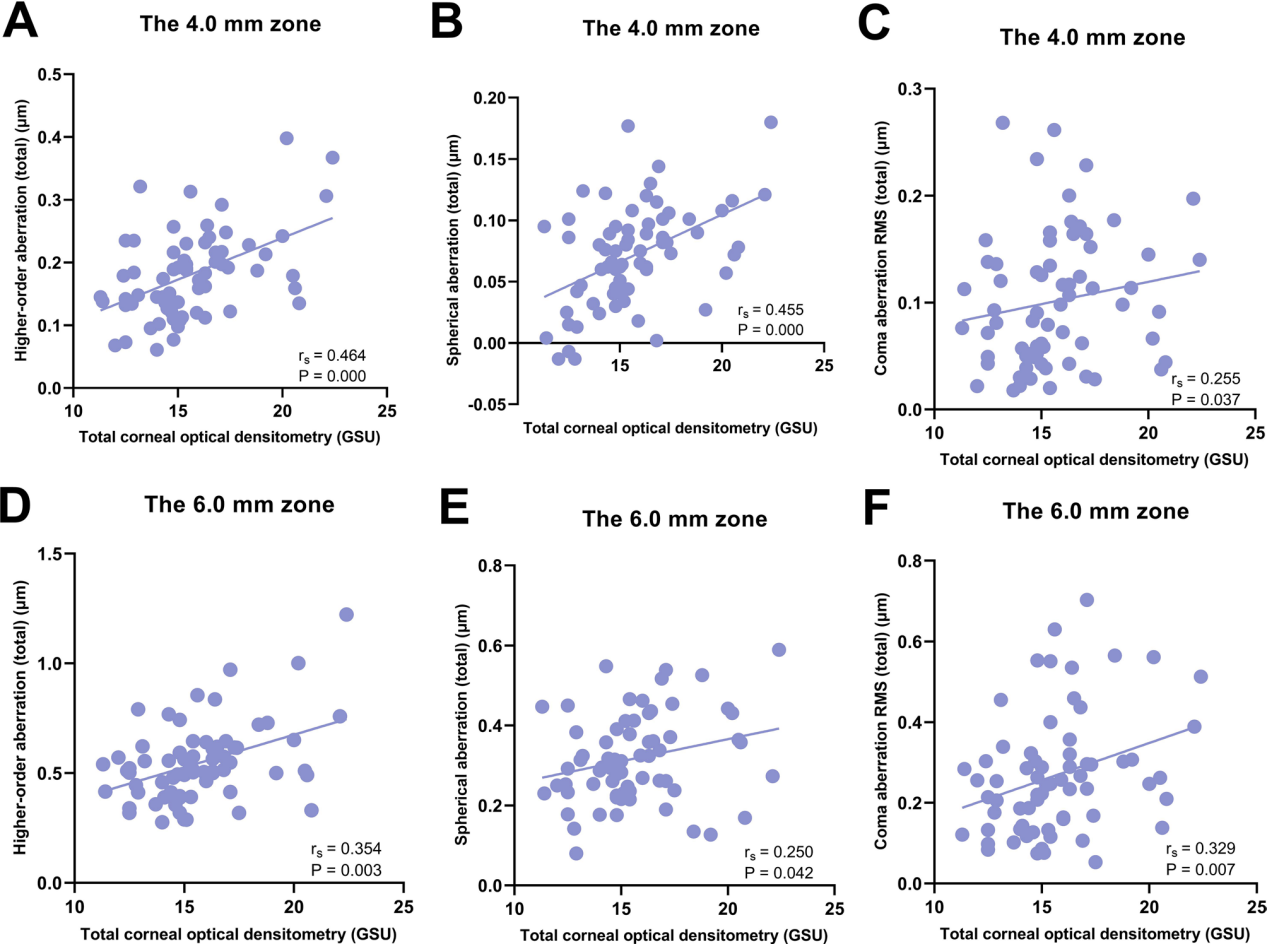
Correlations between total corneal optical densitometry and Higher-order aberrations of the total corneas. Higher-order aberration (total) (**A**), spherical aberration (total) (**B**), and coma aberration RMS (total) (**C**) in the central 4.0 mm diameter zone, as well as higher-order aberration (total) (**D**), spherical aberration (total) (**E**), and coma aberration RMS (total) (**F**) in the central 6.0 mm diameter zone revealed statistically significant positive correlations with total corneal optical densitometry. r_s_ Spearman rank correlation

**Fig. 2:**
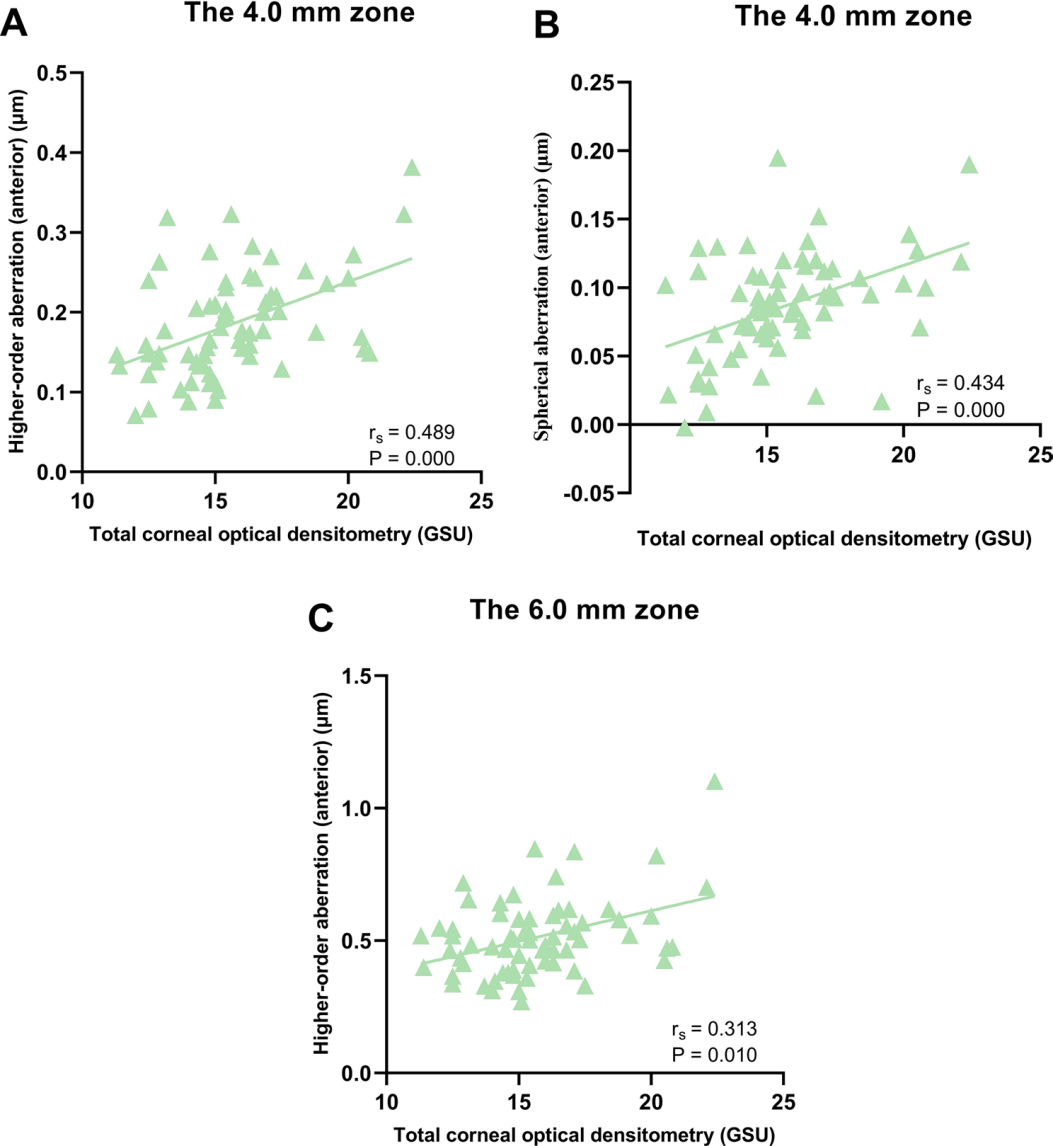
Correlations between total corneal optical densitometry and higher-order aberrations of the anterior corneas. Higher-order aberration (anterior) (**A**), spherical aberration (anterior) (**B**) in the central 4.0 mm diameter zone, and higher-order aberration (anterior) (**C**) in the central 6.0 mm diameter zone revealed statistically significant positive correlations with total corneal optical densitometry. r_s_ Spearman rank correlation

**Fig. 3:**
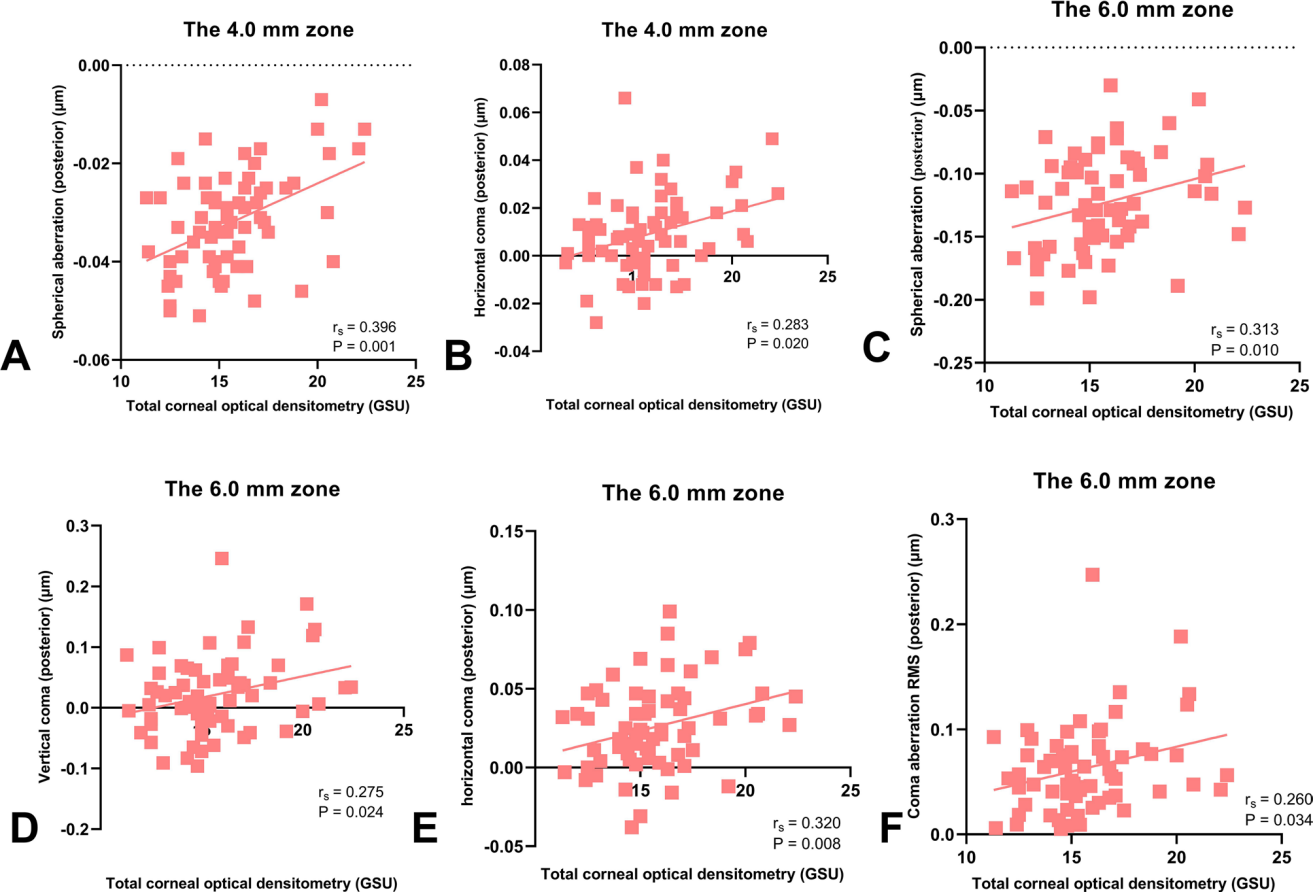
Correlations between total corneal optical densitometry and higher-order aberrations of the posterior corneas. Spherical aberration (posterior) (**A**), horizontal coma (posterior) (**B**) in the central 4.0 mm diameter zone, as well as spherical aberration (posterior) (**C**), vertical coma (posterior) (**D**), horizontal coma (posterior) (**E**), and coma aberration RMS (posterior) (**F**) in the central 6.0 mm diameter zone revealed statistically significant positive correlations with total corneal optical densitometry. r_s_ Spearman rank correlation

**Fig. 4:**
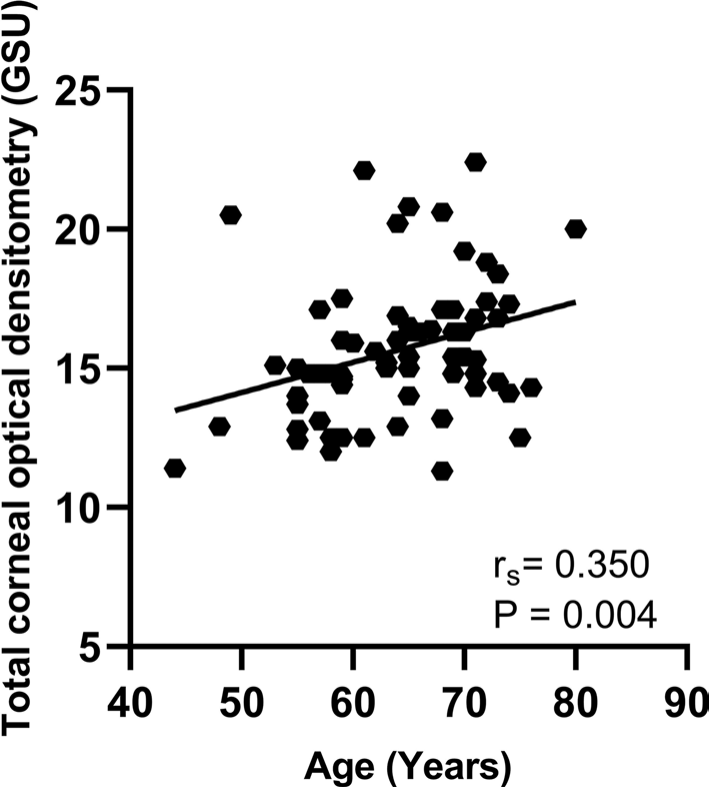
Correlation between total corneal optical densitometry and age

Correlations between total COD and corneal HOAs were presented in Table 3. The data were analyzed and summarized as follows: in the central 4.0 mm diameter zone, total COD measurements exhibited significant positive correlations with HOA, Z40, and Z3 ± 1 RMS values of the total cornea, HOA and Z40 of the anterior cornea, as well as Z40 and Z31 of the posterior cornea; in the 6.0 mm zone, total COD measurements were positively correlated with HOA, Z40, and Z3 ± 1 RMS of the total cornea, HOA of the anterior cornea, as well as Z40, Z3-1, Z31, and Z3 ± 1 RMS of the posterior cornea (*P* < 0.05 for each one). Moreover, the correlations between total COD and COD-related HOAs were shown in scattering graphs (Figs. 1, 2 and 3). In contrast, the Spearman rank correlation analysis revealed no statistically significant correlations between COD and the remaining corneal HOAs (*P* > 0.05 for each one).Furthermore, the study found a statistically positive correlation between total COD and age (r_s_ = 0.350, *P* = 0.004) (Fig. 4).

**Table 3:** Correlations between total corneal optical densitometry and corneal higher-order aberrations

Aberrations	The central 4.0 mm zone		The central 6.0 mm zone	
	Spearman, *r*_s_	*P*	Spearman, *r*_s_	*P*
HOA (total)	0.464	0.000*	0.354	0.003*
Z40 (total)	0.455	0.000*	0.250	0.042*
Z3-1 (total)	0.200	0.104	0.226	0.066
Z31 (total)	-0.007	0.958	-0.028	0.821
Z3 ± 1 RMS (total)	0.255	0.037*	0.329	0.007*
Z3-3 (total)	0.074	0.551	-0.165	0.183
Z33 (total)	-0.008	0.948	0.031	0.806
Z3 ± 3 RMS (total)	0.141	0.255	0.161	0.193
HOA (anterior)	0.489	0.000*	0.313	0.010*
Z40 (anterior)	0.434	0.000*	0.214	0.082
Z3-1 (anterior)	0.227	0.065	0.176	0.155
Z31 (anterior)	-0.078	0.529	-0.099	0.427
Z3 ± 1 RMS (anterior)	0.216	0.079	0.195	0.115
Z3-3 (anterior)	0.017	0.889	-0.167	0.176
Z33 (anterior)	-0.078	0.529	0.078	0.529
Z3 ± 3 RMS (anterior)	0.173	0.161	0.082	0.510
HOA (posterior)	0.025	0.840	0.025	0.838
Z40 (posterior)	0.396	0.001*	0.313	0.010*
Z3-1 (posterior)	0.095	0.444	0.275	0.024*
Z31 (posterior)	0.283	0.020*	0.320	0.008*
Z3 ± 1 RMS (posterior)	0.217	0.077	0.260	0.034*
Z3-3 (posterior)	0.034	0.787	-0.048	0.700
Z33 (posterior)	-0.040	0.746	-0.136	0.273
Z3 ± 3 RMS (posterior)	-0.140	0.258	0.183	0.139

*Significant

## Discussion

This study explored whether COD might be an influencing factor of corneal HOAs and visual quality by correlating the total COD values with corneal HOAs, including total HOA, third-order HOAs, and spherical aberration in clinically clear corneas. To our knowledge, this is the first study to explore the correlations between COD measurements and corneal HOAs. In ophthalmologic examinations, corneal transparency plays a critical role in determining the health of the human eye [19]. Notably, clinical observations reveal that certain corneas, even when subjectively assessed as transparent, may exhibit abnormal variations in COD or other objective indicators of corneal transparency. The variations in COD may have potential implications for visual function, highlighting its significance as a research focus.

Extensive focus has been paid to COD over the past few years. Previous studies have consistently demonstrated that COD values differ significantly across four concentric annular regions and exhibit statistical variations at three distinct depth layers [18]. Moreover, central zones of the cornea exhibited significantly lower COD levels compared to peripheral zones, consistent with clinical observations [18]. Simultaneously, COD levels showed the highest values in the anterior corneal layer and the lowest values in the posterior layer [15, 18]. In previous studies, a comparative analysis of COD and other instruments utilized for measuring corneal transparency was conducted. For instance, in vivo confocal microscopy demonstrated the highest backscatter in the posterior layer [20]. This discrepancy can primarily be ascribed to the non-contact characteristic of the Scheimpflug system, variations in illumination conditions, and differences in image acquisition techniques [15]. It has been demonstrated that disruptions in the regular arrangement and uniform composition of corneal layers contribute to an increase in COD [16]. Similarly, the regional variations in COD may be attributed to the anisotropic nature of corneal microstructure [21]. Therefore, COD can serve as an objective quantification tool for reflecting changes in corneal microstructure and composition.

HOAs of the total cornea are commonly utilized in clinical settings as indicators for assessing human visual quality. In this study, preliminary analyses revealed that COD was statistically significantly positively correlated with corneal total HOA, spherical aberration, and coma aberration of the total cornea in both the central 4.0 mm and 6.0 mm zones. Furthermore, based on the magnitude of the Spearman rank correlation coefficient r_s_, it can be concluded that the degree of correlation ranges from weak to moderate. As is widely recognized, corneal aberrations primarily arise from irregularities in the morphology of the corneal surface, with anterior corneal surface irregularities being the most prevalent. Another significant contributor to corneal aberrations is the non-uniform refractive index resulting from the heterogeneous structure of the cornea. Furthermore, previous studies have shown that pupil size significantly influences optical aberrations [22]. As is well known, total HOA plays an important role in evaluating human visual quality, particularly in the central 4.0 mm optical region of the cornea. The findings regarding the correlation between corneal HOA and COD demonstrated consistency across both the total cornea and the anterior cornea, as well as within the central 4.0 mm and 6.0 mm zones. Among HOAs, spherical and coma aberrations represent the predominant components, and the magnitude of vertical coma aberration exceeds that of horizontal coma aberration [5]. In particular, the impact of spherical aberration on visual quality is typically observed when the pupil is dilated at night under low-light conditions. Therefore, spherical aberration within the central 6.0 mm corneal zone holds greater clinical research significance. In this study, spherical aberration in the 6.0 mm corneal zone showed a statistically significant positive correlation with COD in both the total cornea and the posterior cornea, which can be explained by the substantial influence of the posterior corneal surface on corneal spherical aberration [23]. Additionally, corneal spherical aberration is rotationally symmetric, which reflects corneal asphericity [24]. Furthermore, it is important to note that spherical aberration results from discrepancies in the focal points of central and peripheral rays reaching the retina simultaneously. Consequently, spherical aberration is characterized by a balance between the central and peripheral curvatures of the cornea [5]. The conclusion of previous studies regarding the variations in COD between central and peripheral zones of the cornea supports the correlation between spherical aberration and COD observed in this study. Furthermore, corneal third-order aberrations are non-axial aberrations, reflecting corneal asymmetry and irregularity. The refractive power differs across various regions of the cornea, potentially resulting in coma aberration [23]. Meanwhile, irregularities in corneal surfaces and interfaces between corneal layers increase light scattering, which in turn causes a higher degree of COD [16]. Therefore, the correlations between COD and corneal coma aberration are reasonable and fall within the expected ranges. In the study of the posterior corneal surface, spherical aberration and horizontal coma within the central 4.0 mm zone, as well as spherical aberration, vertical coma, horizontal coma, and coma within the 6.0 mm zone, all exhibited statistically significant positive correlations with COD. Although the contribution of posterior corneal HOAs to overall ocular optical quality is relatively minor, the influence of COD on corneal HOAs should not be disregarded. Based on the research findings, it was hypothesized that an increase in COD could potentially compromise the sphericity, symmetry, and regularity of the cornea, primarily as a result of alterations in corneal microstructure and composition. Furthermore, variations in COD provide valuable assistance in analyzing the abnormal changes in HOAs observed in clinically transparent corneas.

Previous studies have demonstrated that visual symptoms are strongly associated with ocular aberrations, such as halo and glare with spherical aberration, monocular diplopia with coma, and starburst with trefoil [25]. An increase in total HOA may compromise the visual performance [5]. Moreover, spherical aberration is considered to have the most significant impact on the visual performance of all HOAs, and it has been demonstrated that spherical aberration can significantly affect the ability of an optical system to generate images with high contrast and resolution. Therefore, it is reasonable to conclude that variations in COD may potentially affect corneal HOAs, consequently influencing corneal visual quality based on the findings and analysis presented in this study. In addition, Ning et al. [21] employed an OQAS™II device to measure three key ocular parameters: the ocular optical scatter index (OSI), Strehl’s ratio (SR), and modulation transfer function cutoff frequency (MTF_cutoff_), and investigated the correlations between these three parameters and COD. Their results demonstrated a positive correlation between COD and OSI, as well as negative correlations between COD and both MTF_cutoff_ and SR. These findings further underscore the impact of COD on optical quality.

In this study, an increase in COD was observed with aging. In recent years, considerable attention has been directed toward the correlation between COD and age. Cankaya et al. [18] observed a strong correlation between age and COD in all layers and zones, which supported the results of this study. In another study, Tekin et al. [16] demonstrated that COD was statistically associated with endothelial cell properties, thereby providing evidence to support the hypothesis that COD serves as an indicator of corneal endothelium health. Garzón et al. [26] made the point that COD increased with age because of the loss of corneal endothelial cells, which are necessary to maintain corneal transparency [27]. Additionally, other potential factors contributing to this increase may include alterations in corneal biomechanics, rearrangement of the stromal collagen fibers, and degeneration of the corneal limbus, which are commonly found in the elderly population. Similarly, the conclusion that total HOA, coma, and trefoil aberrations of the cornea increase with age has been widely accepted in previous studies [5]. Based on these findings, we hypothesized that the variations in HOAs may potentially be influenced by age-related changes in COD.

This study had several limitations that needed to be addressed. For instance, although measurements of human parameters usually show a normal distribution, this study acquired non-normal distributions for some of the measured ocular parameter values. Therefore, the distributions of COD and different corneal HOAs need to be confirmed with a larger sample size. Additionally, the measurement of aberrations and COD can be influenced by the condition of the tear film [28]. Although eyes diagnosed with dry eye disease were excluded from this study, the potential impact of the tear film on the measurement results cannot be completely disregarded for certain participants.

In conclusion, the optical characteristics of the human ocular system are currently under investigation. Ophthalmologists have attached importance to visual quality for many years, and corneal aberrations analysis plays a vital role in estimating optical quality. In clinical practice, COD has been extensively applied to assess some of the corneal diseases [15], including infectious keratitis [29], keratoconus [30], and corneal dystrophies [31]. The effects of COD on visual quality, however, need to be investigated further. The correlations between COD and corneal HOAs are clinically significant. This finding offers valuable insights for clinicians in their daily practices.

## Supplementary Information

Below is the link to the electronic supplementary material.

Supplementary Material 1

## Data Availability

The data used to support the findings of this study are available from the corresponding authors upon request.
