# Supplementary figures and images for: Corneal optical densitometry in transparent corneas and its correlations with corneal higher-order aberrations
Source: BMC Ophthalmol. 2026 Jan 19;26:80. doi: 10.1186/s12886-025-04583-x (PMC12895795; doi:10.1186/s12886-025-04583-x)

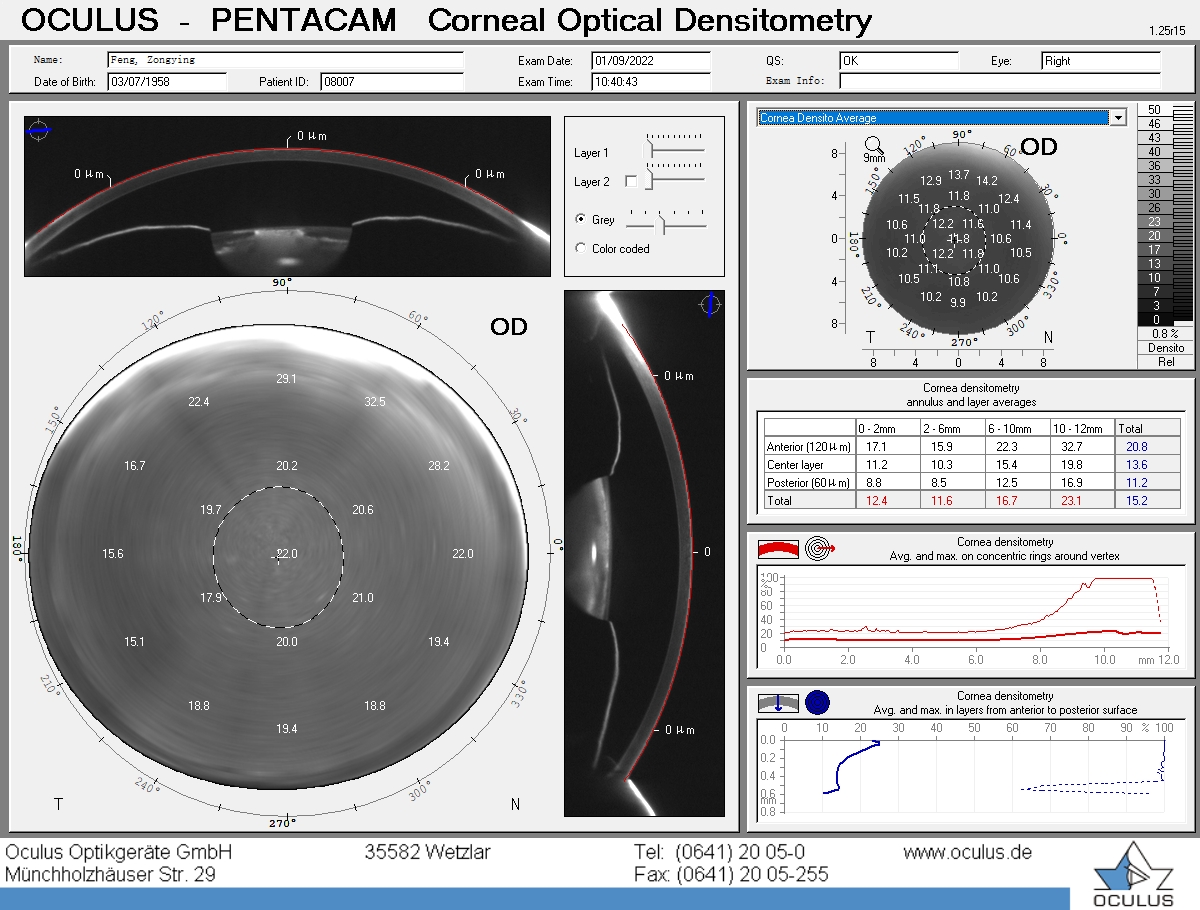


Supplementary figure 1. Screen date output of the corneal optical densitometry measurement

Supplement: Supplementary file 1 — Supplementary Material 1 [file 12886_2025_4583_MOESM1_ESM.doc]
